# Three Abdominal Cases Presenting Some Unusual Features
1Read before the Bristol Medico-Chirurgical Society, May 9th, 1900.


**Published:** 1900-12

**Authors:** A. W. Prichard

**Affiliations:** Senior Surgeon to the Bristol Royal Infirmary


### THREE ABDOMINAL CASES PRESENTING SOME UNUSUAL FEATURES.[1]

BY

A. W. Prichard, M.R.C.S.,

*Senior Surgeon to the Bristol Royal Infirmary.*

---

Three cases involving abdominal operations have come under my care during the last three or four months at the Infirmary presenting some points so much out of the common that I think it is worth while to bring them before your notice:

Case 1.—On a Sunday evening, a little before Christmas, I was summoned to a case of perforated gastric ulcer which had just arrived at the Royal Infirmary. The patient, a domestic servant, æt. 19, had been driven up twenty miles in a cab, and was almost *in extremis*. There was a definite history of perforation occurring at two o'clock in the morning, and she had been allowed no nourishment or stimulant by the mouth since. I operated at 8.30—eighteen hours after the perforation—making a median incision above the umbilicus. Running my finger along the anterior surface of the stomach, I found the laceration to be towards the cardiac end. I made a transverse cut through the left rectus abdominis and exposed the stomach wall. This had two openings in it, a small one and a large one which would easily admit two finger-tips. The condition of the wall of the viscus for a considerable distance round the openings was such that any attempt to get stitches to hold was futile, and the whole area before healthy tissue was reached was too large to be excised. In consistence it seemed like thick, rather rotten, orange peel. After one or two attempts to sew up the holes I abandoned them, and packed iodoform gauze from the

[1] Read before the Bristol Medico-Chirurgical Society, May 9th, 1900.

abdominal wound to the rent in the stomach, leaving a large indiarubber drainage-tube in the centre of the gauze. Previously to this I had mopped out the peritoneum in the immediate neighbourhood, and put a drainage-tube through the flank in front of the left kidney. The patient was in a critical state before the operation, and naturally afterwards her condition was most serious. For some days she was kept alive by nutrient enemata and the wound began to heal. The tube was slowly pushed out and shortened, and I ventured upon giving her some liquid nourishment by the mouth; and at the end of three weeks she was able to digest pretty well, and I had strong hopes of her ultimate recovery. However, her temperature went up, and some pleuritis came on; and a little later, about five weeks after the first operation, a needle passed deeply in above the tenth rib on the left side proved pus to be present. Empyema was diagnosed. She therefore was put under an anæsthetic, and I made an incision into the pleura under the tenth rib. I could find no pus in the pleura, and I excised two inches of the rib and sewed the diaphragm to the parietal wound, and then cut through the diaphragm and successfully passed a sinus forceps into a subphrenic abscess, and put in a drainage-tube. I am sorry to relate that, though the temperature fell, this did not ameliorate all symptoms, and she died three days after the second operation and six weeks from the first. The autopsy showed (1) that there was complete union of the rents in the stomach; (2) that the subphrenic abscess was properly drained, but (3) that there was an abscess in the top of the *right* kidney of which we had no evidence.

Case 2.—The patient, a woman, æt. 37, was admitted at the Infirmary on March 1st, suffering from abdominal symptoms. She had had an attack of what was probably appendicitis nine years before, and had been since habitually very constipated. Now she had been ill for two days, with pain in the front of the abdomen and vomiting. Her bowels had moved slightly in the morning, and she was somewhat under the influence of morphia. After trying the effect of an oil and a simple enema, which was unsatisfactory, I thought an immediate operation advisable. Five hours after admission, I opened the abdomen in the middle line below the umbilicus, and at once came down upon the seat of trouble. There was a mass of matted intestine near the middle line, just under the abdominal wound. There was a very long appendix, thin and isolated, like a band, stretching from the right iliac region to this matting, where the intestine was firmly adherent to its head. It was difficult to unravel this, so firm were the adhesions, so I cut off the appendix at its root and treated the stump with pure carbolic acid. On attempting again to free the matted coil, I found that in one part it was quite impossible. It was as if the head of the appendix had become attached to the mesentery of a loop of small intestine, and the peritonitic bands surrounded the proximal end of the loop so tightly that the intestine was very much compressed; the distal end of the loop would permit of invagination of the little finger, and could be stretched. I could not free the bands round the proximal end, and slightly tore the intestinal wall in my attempt, so I put enterectomy clamps on the bowel and excised about two inches of intestine with a V-shaped piece of mesentery. I sewed the two ends of the bowel carefully together with two continuous silk Lembert sutures, one from each side of the mesenteric attachment, and tied the two sutures together on the side of the bowel opposite the mesentery. I also sewed up the V-cut in the mesentery by silk, passing the stitches right through to arrest some bleeding. I will not trouble you with details of after-treatment. The patient made a good recovery, though there was some suppuration three or four days after in the abdominal wound, cultures from which showed the presence of bacterium coli, and I was afraid a leak had taken place and a fistula might form. However, it all healed perfectly soundly, and the patient was able to take fish or fowl after the eighteenth day. She was discharged on April 14th, just over six weeks from the operation, able to eat anything, and having regular and painless action of the bowels.

CASE 3.—A woman, æt. 53, was sent to me April 4th by Dr. Browne, of Brynmawr, Breconshire, for treatment of an ovarian tumour. The patient had noticed the swelling for about one year, and the menopause had occurred five or six years ago. I give great credit to Dr. Browne for his diagnosis, as I think all the Infirmary surgeons from external examination were of opinion that the case was one of fibroid tumour of the uterus. One of the resident staff remarked that in three cases of solid ovarian tumour that he had seen there was effusion in the right pleura, and there was effusion in the present instance. The operation was simple, though I had to enlarge the wound to above the umbilicus, so great was the mass. The tumour was a large single solid unadherent ovarian, and was easily removed, the pedicle being tied with silk. I brought the abdominal wound together with silkworm-gut sutures taken through skin, muscle, peritoneum,—peritoneum, muscle, skin. Such has been my custom always, and I have never seen any bad result from the plan. All went well for ten days, when I was summoned down to the Infirmary in the middle of the night, because the patient had signs of intestinal obstruction. She was vomiting, but not distended, and an enema brought about no action of the bowels, though she passed some flatus. I ordered a turpentine enema, and next morning, as there was no relief, I opened the middle of the old cicatrix, and found the intestine matted and firmly adherent to the parietes at the wound. I separated all this with the handle of the scalpel, and brought down a piece of omentum and spread it out to lie between the bowels and the abdominal wall, and sutured the wound in the same way as before. Since then she has had no bad symptoms, and is now up and well, and going home.

In the gastric ulcer case, the points I wish to bring forward are :—First, that the perforation healed without the edges being brought together, but simply by gauze drainage down to the outside of the laceration in the stomach; and, secondly, the method adopted of getting at the subphrenic abscess. After removing a piece of rib—the diaphragm at this part lying close to the thoracic wall—there was plenty of room to attach the diaphragm to the wall, and so make the opening for a drain safe from contaminating the pleural cavity.

In the enterectomy case, the reason I chose close stitching of the peritoneal covering of the bowel in preference to the use of Murphy's button, or any other treatment, was, because I thought I could do it more quickly, and every minute was of importance in the state the patient was then in. If two needlefuls are used, started from a firm knot at the mesenteric attachment and tied together on the opposite point of the bowel, the proceeding can be done very quickly, the chief danger being in making the circle of stitches too tight.

In the last case is there any possible connection between effusion in the pleura, and solid ovarian disease? I cannot see that there is. Secondly, is it not the safest method of closing the abdominal wound to include the peritoneum in the suture? In my opinion sewing only the skin and muscles, and leaving a double edge of cut peritoneum free towards the cavity, is much more likely to give a chance of adhesion to the bowel than when the peritoneum is neatly kept forwards into the wound by the sutures. Do the silkworm-gut sutures passing into the cavity give a special chance for the intestine to become adherent to them?

